# Psychotherapy as a treatment modality for psychiatric disorders: Perceptions of general public of Karachi, Pakistan

**DOI:** 10.1186/1471-244X-9-37

**Published:** 2009-06-15

**Authors:** Abdul Mueed Zafar, Ali Jawaid, Hiba Ashraf, Ambreena Fatima, Rubina Anjum, Salah U Qureshi

**Affiliations:** 1 Aga Khan University Medical College, Karachi, Pakistan; 2 Department of Neurology, Baylor College of Medicine, Houston, USA; 3 Department of Family Medicine, Aga Khan University, Karachi, Pakistan

## Abstract

**Background:**

Psychiatric disorders affect about 450 million individuals worldwide. A number of treatment modalities such as psychotropic medications, psychotherapy and electroconvulsive therapy can be used to treat these disorders. Attitudes of general public play a pivotal role in effective utilization of mental health services. We explored the perceptions of general public of Karachi, Pakistan regarding psychotherapy.

**Methods:**

A cross-sectional study was conducted in Karachi, Pakistan during July-August, 2008. A three-step sampling strategy and a structured questionnaire were employed to survey knowledge and perceptions of adult general public about psychotherapy. Descriptive statistics were used for baseline characteristics. Logistic regression models were used to investigate any significant associations between baseline characteristics of the participants and their perceptions.

**Results:**

The study sample comprised of 985 individuals (536 males; 531 financially independent) with an average age of 36.7 years (SD 13.54 years) and 12.5 years (SD 3.09 years) of education were included. Majority (59.4%; n = 585) claimed to be aware of psychotherapy as a treatment option for psychiatric disorders but 47.5% of these (n = 278/585) failed to identify its correct definition. Concerns voiced by the participants about psychotherapy included stigma (48.7%) and breech in confidentiality (39.5%); 60.7% opined it cost effective and 86.5% favored its use as an adjuvant modality. A preference for psychotherapy as the treatment strategy for psychiatric disorders was demonstrated by 46.6% (n = 459/985). Younger, more educated, financially independent and female participants were more likely to prefer psychotherapy as were those who deemed it cost effective.

**Conclusion:**

Positive attitudes regarding the acceptability, clinical utility and cost-effectiveness of psychotherapy were observed in a sample representative of general public of Karachi, Pakistan. These findings highlight its potential utility for devising pragmatic mental health strategies in the face of limited resources.

## Background

Psychiatric disorders rank among the leading causes of morbidity worldwide. They affect about 450 million individuals and the burden is projected to track an upward trend. [1,2] These disorders account for 12.3% of Disease Adjusted Life Years (DALYs) globally. [1] This becomes highly pertinent in context of countries like Pakistan where the mean overall prevalence of anxiety and depression is around 34% with estimates as high as 66% for certain population subsets, the government funded health facilities are scarce and 98% of private health expenditure is paid out-of-pocket by the patient or his/her family. [3-6]

Multiple options, such as pharmacotherapy, psychotherapy and electroconvulsive therapy (ECT), are available for the management of psychiatric disorders. Nevertheless, even in affluent western societies only a third of psychiatric patients receive appropriate treatment. [2] Perceptions and preferences of patients, caregivers and general public are instrumental in increasing the utilization of mental health services. [7-13] Positive attitudes towards a particular treatment modality have been associated with greater acceptability and compliance whereas reservations about its efficacy and cost-effectiveness usually have lead to non-compliance. [2,11,14-16] In order to be successful, psychiatric treatment strategies should be tailored to the needs, resources, perceptions and preferences of specific socio-demographic groups. [1,7,9]

A thorough MEDLINE search reveals that general public across different countries appears to have a preference for psychotherapy over other psychiatric treatment modalities. [10,12-14,17-26] We explored into the perceptions of the general public of Karachi, the largest metropolitan of Pakistan, towards psychotherapy as a psychiatric treatment modality and investigated any association between these perceptions and age, gender, educational level or occupational status of the participants. Preferences pertaining to the modality and setting for treatment of psychiatric diseases were also documented during this survey.

## Methods

A cross-sectional survey was conducted in Karachi, from July 2007 through August, 2008. The metropolitan comprises about a tenth of the total populace of Pakistan and is politically divided into 18 towns. Each town is further divided into 7 to 13 Union Councils (UC). [27] A three step sampling strategy was employed. At the first step three towns were selected through random draws. At the second step, three Union Councils were identified within each of the three towns, in the same manner. From each of the nine selected UCs (UC 2, 3 and 12 in *Gulshan-e-Iqbal *town; UC 4, 7 and 11 in *Jamshed *town; UC 1, 2 and 7 in *Malir *town), a non-probability convenient sample of adults (age 18 years and above) were requested to participate in the study. Individuals meeting any of the following criteria were excluded: 1) affiliation with medical profession i.e. doctors, nurses and medical students 2) history of a psychiatric illness 3) use of psychotropic drugs within the preceding one month.

Ethical review was waived by the Ethical Review Committee of Aga Khan University for the survey as any identifying information was not obtained from the participants. The study was conducted in compliance with the 'Ethical principles for medical research involving human subjects' of Helsinki Declaration. A verbal informed consent was taken from all the participants.

A structured questionnaire was developed in '*Urdu*' (national language of Pakistan) after a thorough review of literature and consultations with two psychiatrists. The questionnaire was validated linguistically and pre-tested on a subset of the target population (n = 25, not included in analysis) to ensure conceptual clarification, consistency of responses and feasibility of administration. Participants' age (in years), gender, level of education (years of schooling) and financial status (independent/dependant) were recorded in the first part of the questionnaire (Additional file 1). They were then asked about their familiarity with different psychiatric treatment modalities (viz. Pharmacotherapy, Psychotherapy and ECT). A skip pattern was used at this point; only those asserting an awareness of psychotherapy were asked to identify its true definition [28] out of four choices. The perceptions of this subset regarding psychotherapy were also explored using dichotomous response variables (Agree/disagree). Finally, all the participants were asked to identify the first person they would consult upon experiencing symptoms of anxiety or depression. They were also requested to state the treatment modality (psychotropic medications, psychotherapy or ECT) and treatment setting (Psychiatric institute, General Hospital, Community health clinic or Home) they would prefer for themselves/their family in the event of suffering from a psychiatric condition. The final questionnaire was administered by the authors (AMZ, AF, HA, RA).

The data were entered, validated and analyzed using SPSS version 16.0. Descriptive statistics were employed for baseline characteristics as well as perceptions of the participants. Logistic regression models were used to investigate any relationship between the perceptions and baseline characteristics. For all analysis level of significance (α) was set as 0.05

## Results

From the 1704 individuals approached, 1066 consented to participate in the study, resulting in a modest response rate of 62.6%. Seventeen participants divulged a history of psychiatric illness (Depression, n = 13; Not specified, n = 4). Fifty-two reported use of psychotropic drugs (tranquilizers/sedatives, n = 52) in the preceding month. Twelve individuals did not complete the interview.

Data from 985 respondents were included in final analysis. The study sample comprised 536 (54.4%) males and 449 (45.6%) females with an overall mean age of 36.7 years (SD 13.54 years; Range 18–86 years) and an average 12.5 years (SD 3.09; Range 5–16) of education. With respect to financial status, 53.9% (n = 531) reported being independent whereas 46.1% (n = 454) categorized themselves as dependant.

Among the psychiatric treatment modalities, highest awareness was documented for Pharmacotherapy (87.0%; n = 857) followed by psychotherapy (59.4%; n = 585) and ECT (45.1%; n = 444). On the other hand, 47.5% of those who claimed to be aware of psychotherapy (n = 278/585) failed to identify its correct definition. The perceptions regarding psychotherapy as well as their comparisons across age, gender, education and occupational status are elaborated in Additional file 2. **The majority supported the use of psychotherapy as an adjuvant to pharmacotherapy (80.5%) and considered it a cost effective modality (60.7%). Less than half of the respondents were of the view that stigma is linked to the utilization of psychotherapy services**. Psychiatric treatment seeking practices of the participants are given in Additional file 3. **Eighty-eight percent of the participants reported that they would first consult either a psychiatrist or a general practitioner in case someone experiences symptoms of anxiety or depression. The study sample had an almost equivalent preference for pharmacotherapy (46.6%) and psychotherapy (48.7%) for the treatment of psychiatric diseases**. To identify the predictors of choice for psychotherapy as a treatment modality, a logistic regression model was built. The pseudo R-squared of the model was 0.374. (Additional file 4) **A significantly greater preference for psychotherapy was noticed among younger, females, more educated and financially independent participants. Similar was the case for respondents who were better aware of this modality and who supported its use as an adjuvant to pharmacotherapy. (p < 0.05)**

## Discussion

The results of this study suggest that general public of the largest city of Pakistan perceive psychotherapy as a clinically effective, cost efficient and acceptable modality for management of mental ailments. Psychotherapy is a widely used psychiatric treatment modality. A huge body of literature supports its role as an adjuvant to medications in multiple psychiatric conditions. [19,25,26] Even as a monotherapy, it has been found to be more effective than medications in certain variants of depression. [18,23]

Explorations in diverse cultural settings have shown that, in addition to clinical effectiveness, popularity of any treatment modality is an important determinant of its optimal utilization. [10,11,29-31] Majority of participants in the current study claimed to be aware of psychotherapy. However, a sizable fraction of these was unable to identify the correct definition. Also, those with correct knowledge were more likely to opt in favor of psychotherapy. This identifies a need for augmenting awareness about psychiatric treatment options available in Pakistan as well as for ascertaining the reliability of the sources of this information.

A key finding of this survey is that only a minority linked the use of psychotherapy with stigma. A breech in confidentiality was concern of an even smaller fraction. Cost effectiveness and clinical efficacy are two other factors that have been identified to be imperative for optimal utilization of mental health services in South Asia. [11] In the current study, a large proportion deemed psychotherapy cost effective and corroborated its application as a primary/adjuvant therapeutic modality for psychiatric disorders. Our observations of a positive public attitude towards psychotherapy concur with the previous reports of general public's perceptions regarding this treatment modality in three different continents. [10,17,20,24] These findings bring forth psychotherapy as a potentially successful and acceptable psychiatric treatment modality in Pakistan. [29-32]

It has been observed in the West that, while the general public has a reluctance towards the use of psychotropic medications, the acceptance for psychotherapy remains high. [12,14,21,22] A general predilection for psychotherapy as the primary treatment modality in psychiatric illnesses has been demonstrated among lay population of both developed and developing countries. [10,13,17] We observed an almost equivalent preference for psychotherapy and psychosomatic medications as the primary treatment modality among lay public of Karachi. This divergence from previous observations could be attributed to the fact that a large proportion of the population we surveyed did not have adequate awareness regarding utility of psychotherapy in treatment of psychiatric disorders. Although it may be speculated that this owes to a scarcity of psychotherapy services in Pakistan, a targeted exploration will make the picture more vivid.

When asked about the preferred care provider and setting of treatment, the participants identified psychiatrists and psychiatric institutes most frequently. Similar preferences for specialized psychiatric services have also been demonstrated in other countries. [7,13] But an interesting finding in our study was that the General Practitioner was a close second preference for initial consultation in case of symptoms suggestive of psychiatric disorder. Although a low preference for psychiatric treatment at community health clinic limits the potential implications of this finding, it does allude towards the GP as a potential resource for mental health screening; perhaps even treatment of selected disorders. [33]

Treatment for psychiatric disorders remains a neglected avenue in Pakistan. The total number of psychiatrists for the large country populace is only 300 and the mental health budget comprises a minute fraction (0.4%) of total health budget. [6,34] A pressing need for development of psychiatric treatment services in the face of limited resources makes the opinions of lay public all the more important while strategizing for this expansion.

There are some limitations that must be considered before generalizing the findings of our study to other populations. Firstly, an unassuming response rate raises the possibility of a selection bias that may be attributable to a general reluctance towards the discussion of psychiatric health issues in Pakistan. Secondly, the sample comprised of individuals with an urban background and a high level of education. A disparity of opinions among the rural and urban population is strongly speculated in the background of high levels of illiteracy among the rural population as well as their strong reliance on traditional faith healers (*shamans*) for treatment of psychiatric disorders. [3-5,34] Thus, adoption of a cautious approach while drawing any implications from the results of this survey is recommended.

## Conclusion

Positive attitudes regarding the acceptability, clinical utility and cost-effectiveness of psychotherapy were documented in a sample of the lay public of Karachi, Pakistan. These findings open avenues for further targeted research on the issue and may contribute for strategizing the provision of psychiatric services in Pakistan.

## Competing interests

The authors declare that they have no competing interests.

## Authors' contributions

AMZ conceived the idea, designed the study, collected data, performed statistical analysis, and drafted the manuscript. AJ conceived the idea, designed the study, and drafted the manuscript. HA collected data, performed statistical analysis and critically reviewed the manuscript. AF collected data, coordinated the study, and critically reviewed the manuscript. RA data, coordinated the study, and critically reviewed the manuscript. SUQ conceived the idea and drafted the manuscript. All authors have read and approved the final manuscript.

## Pre-publication history

The pre-publication history for this paper can be accessed here:



## Supplementary Material

Additional file 1 **Questionnaire**. English version of the administered questionnaire (Authors' translation). Click here for file

Additional file 2 **Table 1**. Comparison of knowledge and perceptions regarding psychotherapy among study participants* with varying age, level of Education, gender (male vs. female) and financial status (Independent vs. dependant). Click here for file

Additional file 3 **Table 2R**. Practices of participants pertaining to psychiatric treatment seeking. Click here for file

Additional file 4 **Table 3**. Logistic Regression Model elaborating the predictors of choice of psychotherapy as a psychiatric treatment modality among study participants.* Click here for file
