# Supplementary material for: Psychotherapy as a treatment modality for psychiatric disorders: Perceptions of general public of Karachi, Pakistan
Source: BMC Psychiatry. 2009 Jun 15;9:37. doi: 10.1186/1471-244X-9-37 (PMC2702376; doi:10.1186/1471-244X-9-37)
Supplement: Additional file 1 — Questionnaire. English version of the administered questionnaire (Authors' translation). [file 1471-244X-9-37-S1.doc]

# Psychotherapy as a Treatment Modality for Psychiatric Disorders: Perceptions of General Public of Karachi, Pakistan.

We are a group of researchers affiliated with Aga Khan University, Karachi, Pakistan and Baylor College of Medicine, Houston, Texas, USA. We are conducting a study to explore the opinions of general public of Karachi about a method of treating psychiatric disorders. We would appreciate if you consent to participate in this study. We would request you to spare about 15 minutes of your valuable time to answer the following questions. All information obtained will be used for scientific purposes only. Any individual identifying information of the participants will be kept confidential. You would have the right to withdraw during any phase of the study. We would like to start with your consent.

Consent granted:  Yes  No

Name and Signature (optional): _______________________

Q1. Are you below 18 years of age?  Yes  No

Q2. Are you a doctor, nurse or medical/nursing student?  Yes  No

Q3. Have you ever been diagnosed with any psychiatric illness?  Yes  No

If yes, please specify (*optional*)___________________

Q4. Have you used psychotropic drugs within the last month?  Yes  No

If yes, please specify (*optional*) ___________________

*If response to any of Q1-4 is ‘****Yes****’, thank the respondent for his/her valuable time and conclude the interview. Otherwise, please proceed to Q5.*

Q5. Gender:  Male  Female

Q6. Age: ___________ years

Q7. Education: ______ years of schooling

Q8. Financial status:  Independent  Dependant

Q9. Are you familiar with the following psychiatric treatment modality?

Q9.1 Psychotropic medications  Yes  No

Q9.2 Psychotherapy  Yes  No

Q9.3 Electro-convulsive therapy  Yes  No

*If the response to Q9.2 is ‘****No****’ please skip to Q18.*

*If the response to Q9.2 is ‘****Yes****’, please proceed to Q10.*

Q10. Please choose the single best option,

Psychotherapy is a psychiatric treatment modality based on …

1. ventilating negative emotions in the presence of a qualified therapist.
2. verbal communication or discussion with a qualified therapist.
3. sleep and relaxation under supervision of a qualified therapist.
4. mental exercises under direction of a qualified therapist

*For Q11-17, please state whether you agree or disagree with the given statement.*

Q11. Stigma is associated with the use of Psychotherapy.  Agree  Disagree

Q12. Patient confidentiality may be breeched during Psychotherapy.  Agree  Disagree

Q13. Psychotherapy can bring about permanent changes in patient’s personality.  Agree  Disagree

Q14. Psychotherapist may start controlling patient’s mind.  Agree  Disagree

Q15. Psychotherapy is a cost effective treatment.  Agree  Disagree

Q16. Psychotherapy should be the primary treatment modality.  Agree  Disagree

Q17. Psychotherapy should be an adjuvant to pharmacotherapy.  Agree  Disagree

Q18. In the event someone has symptoms of anxiety or depression, I would ***first*** consult (*Please select one*)

1. Psychiatrist
2. General practitioner
3. Faith healer
4. Alternative medicine practitioner
5. Other (*please specify*) ________

Q19. If a member of my family/ myself is diagnosed with a psychiatric illness, I would ***prefer*** the following treatment modality (*Please select one*)

1. Psychotropic medications
2. Psychotherapy
3. Electro-convulsive therapy

Q20. If a member of my family / myself is diagnosed with a psychiatric illness, I would ***prefer*** that treatment be instituted at (*Please select one*)

1. Psychiatric Institute
2. Home
3. General hospital
4. Community Health Clinic

**~ ~ Thank you ~ ~**
