# Supplementary material for: Psychotherapy as a treatment modality for psychiatric disorders: Perceptions of general public of Karachi, Pakistan
Source: BMC Psychiatry. 2009 Jun 15;9:37. doi: 10.1186/1471-244X-9-37 (PMC2702376; doi:10.1186/1471-244X-9-37)
Supplement: Additional file 2 — Table 1. Comparison of knowledge and perceptions regarding psychotherapy among study participants* with varying age, level of Education, gender (male vs. female) and financial status (Independent vs. dependant). [file 1471-244X-9-37-S2.doc]

**Table 1: Comparison of knowledge and perceptions regarding psychotherapy among study participants* with varying age, level of Education, gender (male vs. female) and financial status (Independent vs. dependant)**

|  |  |  |  |  |  | Gender | |  | Financial status | |  |
| --- | --- | --- | --- | --- | --- | --- | --- | --- | --- | --- | --- |
|  | Overall  (n=585) | Age, years | | Education, years | | Male  (n=300) | Female  (n=285) |  | Independent  (n=300) | Dependant  (n=285) |  |
|  | n (%) | Mean (SD) | OR  (95% CI)^†^ | Mean (SD) | OR  (95% CI)^†^ | n (%) | n (%) | OR  (95% CI)^†^ | n (%) | n (%) | OR  (95% CI)^†^ |
| Identified True definition | 307 (52.5) | 37.4 (14.3) | 1.1  (1.1-1.2)^‡^ | 13.1 (3.0) | 1.0  (0.9-1.0) | 174 (58.0) | 133 (46.7) | 5.1  (2.8-9.4)^‡^ | 149 (49.7) | 158 (55.4) | 0.2  (0.1-0.4)^‡^ |
| Stigma is associated with it | 285 (48.7) | 35.1 (12.9) | 1.0  (0.9-1.1) | 13.4 (2.6) | 1.0  (1.0-1.1) | 158 (52.7) | 127 (44.6) | 3.0  (1.7-5.1)^‡^ | 139 (46.3) | 146 (51.2) | 0.4  (0.2-0.6)^‡^ |
| Patient confidentiality may be breeched | 231 (39.5) | 38.8 (13.4) | 1.1  (1.1-1.2)^‡^ | 13.3 (2.5) | 1.0  (0.9-1.1) | 118 (39.3) | 113 (39.6) | 1.0  (0.6-1.6) | 119 (39.7) | 112 (39.3) | 0.9  (0.6-1.6) |
| It changes personality permanently | 351 (60.0) | 36.3 (13.9) | 1.0  (0.9-1.0) | 13.3 (2.6) | 1.0  (0.9-1.1) | 156 (52.0) | 195 (68.4) | 0.2  (0.1-0.4)^‡^ | 176 (58.7) | 175 (61.4) | 2.5  (1.4-4.4)^‡^ |
| Psychotherapist starts controlling patient’s mind | 267 (45.6) | 36.5 (15.0) | 1.0  (0.9-1.0) | 13.2 (2.4) | 1.0  (0.9-1.1) | 110 (36.7) | 157 (55.1) | 0.5  (0.3-0.8)^‡^ | 115 (38.3) | 152 (53.3) | 0.8  (0.5-1.4) |
| It is a cost effective treatment | 355 (60.7) | 34.0 (11.3) | 0.9  (0.8-0.9)^‡^ | 13.5 (2.3) | 1.0  (0.9-1.1) | 188 (62.7) | 167 (58.6) | 1.0  (0.6-1.6) | 189 (63.0) | 166 (58.2) | 1.3  (0.8-2.2) |
| It should be the primary treatment modality | 331 (56.6) | 35.4 (13.9) | 1.0  (0.9-1.0) | 12.9 (2.9) | 0.9  (0.8-0.9)^‡^ | 146 (48.7) | 185 (64.9) | 0.8  (0.5-1.4) | 141 (47.0) | 190 (66.7) | 0.6  (0.3-0.9)^‡^ |
| It should be an adjuvant to pharmacotherapy | 506 (86.5) | 35.6 (12.4) | 0.9  (0.9-1.0) | 13.5 (2.6) | 1.1  (1.1-1.2)^‡^ | 256 (85.3) | 250 (87.7) | 0.8  (0.4-1.8) | 256 (85.3) | 250 (87.7) | 0.8  (0.4-1.7) |

* Only for participants aware of psychotherapy (n=585)

† OR= exp(β); Logistic Regression Model; Dependent variable= Agreement to the perception; Independent variables= Age (covariate), Gender (factor), Education (covariate), Financial status (Factor); Reference categories= Female for gender and Dependant for financial status

‡ Significant; α<0.05
