# Supplementary material for: Psychotherapy as a treatment modality for psychiatric disorders: Perceptions of general public of Karachi, Pakistan
Source: BMC Psychiatry. 2009 Jun 15;9:37. doi: 10.1186/1471-244X-9-37 (PMC2702376; doi:10.1186/1471-244X-9-37)
Supplement: Additional file 3 — Table 2R. Practices of participants pertaining to psychiatric treatment seeking. [file 1471-244X-9-37-S3.doc]

**Table 2 - Practices of participants pertaining to psychiatric treatment seeking**

|  | % (n) |
| --- | --- |
| In the event someone has symptoms of anxiety or depression I would ***first*** consult… | |
| Psychiatrist | 44.6 (439) |
| General practitioner | 43.4 (428) |
| Faith healer/ *Shaman* | 8.6 (85) |
| Alternative medicine practitioner | 3.4 (33) |
| If a member of my family is diagnosed with a psychiatric illness, I would ***prefer*** the following treatment modality… | |
| Pharmacotherapy | 48.7 (480) |
| Psychotherapy | 46.6 (459) |
| ECT | 4.7 (46) |
| If a member of my family is diagnosed with a psychiatric illness, I would ***prefer*** that treatment be instituted at… | |
| Psychiatric Institute | 57.3 (564) |
| Home | 22.0 (217) |
| General hospital | 16.0 (158) |
| Community Health Clinic | 4.7 (46) |
| **Total** | **100.0 (985)** |
