# Supplementary material for: Psychotherapy as a treatment modality for psychiatric disorders: Perceptions of general public of Karachi, Pakistan
Source: BMC Psychiatry. 2009 Jun 15;9:37. doi: 10.1186/1471-244X-9-37 (PMC2702376; doi:10.1186/1471-244X-9-37)
Supplement: Additional file 4 — Table 3. Logistic Regression Model elaborating the predictors of choice of psychotherapy as a psychiatric treatment modality among study participants.* [file 1471-244X-9-37-S4.doc]

**Table 3: Logistic Regression Model elaborating the predictors of choice of psychotherapy as a psychiatric treatment modality among study participants.***

|  | B (SE) | Wald | OR (95% CI)^†^ | Sig. |
| --- | --- | --- | --- | --- |
| Age (Years) | -0.03 (0.01) | 14.560 | 0.97 (0.95 to 0.98) | 0.00 |
| Male Gender | -2.01 (0.42) | 23.295 | 0.13 (0.06 to 0.30) | 0.00 |
| Education (Years) | 0.38 (0.05) | 51.805 | 1.46 (1.32 to 1.62) | 0.00 |
| Financially Independent | 1.40 (0.40) | 11.935 | 4.04 (1.83 to 8.91) | 0.00 |
| Identified True definition | 1.11 (0.27) | 17.024 | 3.04 (1.79 to 5.15) | 0.00 |
| Stigma is associated with it | 0.72 (0.28) | 6.730 | 2.05 (1.19 to 3.54) | 0.01 |
| Patient confidentiality may be breeched | 0.11 (0.25) | 0.196 | 1.12 (0.68 to 1.84) | 0.66 |
| It changes personality permanently | -0.61 (0.29) | 4.327 | 0.54 (0.30 to 0.96) | 0.04 |
| Psychotherapist starts controlling patient’s mind | 0.49 (0.28) | 3.086 | 1.64 (0.94 to 2.85) | 0.08 |
| It is a cost effective treatment | 1.02 (0.26) | 15.018 | 2.78 (1.66 to 4.65) | 0.00 |
| It can be used as the primary treatment modality | -0.28 (0.26) | 1.181 | 0.76 (0.46 to 1.25) | 0.30 |
| It can be used as an adjuvant to pharmacotherapy | -1.64 (0.47) | 12.162 | 0.19 (0.08 to 0.49) | 0.00 |
| Constant | -1.61 (0.94) | 2.955 | 0.20 | 0.09 |

* Only for participants whose opinions were recorded (n=585)

† OR= exp(β); Logistic Regression Model; Dependent variable= Choice of psychotherapy; Independent variables= Age (covariate), Gender (factor), Education (covariate), Financial status (factor), Agreement to a perception (factor); Reference categories= Female for gender, Dependant for financial status and Disagree for perceptions
